# Lower-Body Power, Body Composition, Speed, and Agility Performance among Youth Soccer Players

**DOI:** 10.3390/sports12050135

**Published:** 2024-05-16

**Authors:** Cíntia França, Élvio Rúbio Gouveia, Francisco Martins, Andreas Ihle, Ricardo Henriques, Adilson Marques, Hugo Sarmento, Krzysztof Przednowek, Helder Lopes

**Affiliations:** 1 Department of Physical Education and Sport, University of Madeira, 9020-105 Funchal, Portugal; erubiog@staff.uma.pt (É.R.G.); joao.martins@staff.uma.pt (F.M.); hlopes@staff.uma.pt (H.L.); 2 LARSYS, Interactive Technologies Institute, 9020-105 Funchal, Portugal; 3 CIPER, Faculty of Human Kinetics, University of Lisbon, 1495-751 Lisbon, Portugal; amarques@fmh.ulisboa.pt; 4 Center for the Interdisciplinary Study of Gerontology and Vulnerability, University of Geneva, 1227 Carouge, Switzerland; andreas.ihle@unige.ch; 5 Department of Psychology, University of Geneva, 1227 Carouge, Switzerland; 6 Swiss Center of Expertise in Life Course Research LIVES, 1227 Carouge, Switzerland; 7 Marítimo da Madeira–Futebol SAD, 9020-208 Funchal, Portugal; ricardo.henriquesfut@gmail.com; 8 ISAMB, Faculty of Medicine, University of Lisbon, 1649-020 Lisbon, Portugal; 9 Research Unit for Sport and Physical Activity (CIDAF), Faculty of Sports Sciences and Physical Education, University of Coimbra, 3000-115 Coimbra, Portugal; hg.sarmento@gmail.com; 10 Institute of Physical Culture Sciences, Medical College, University of Rzeszów, 35-959 Rzeszów, Poland; krprzednowek@ur.edu.pl; 11 Research Center in Sports Sciences, Health Sciences, and Human Development (CIDESD), 5000-801 Vila Real, Portugal

**Keywords:** adolescent, age, body fat, fat-free mass, football, vertical jumps

## Abstract

Speed and agility have been described as crucial abilities for soccer players. The purpose of this study was to analyze, in detail, the variance in speed and agility tasks explained by lower-body power after controlling for age and body composition. The participants were 96 male soccer players aged 16.1 ± 1.6 years. Body composition (stature, body fat percentage—BF%, body mass, and fat-free mass—FFM), lower-body power (countermovement jump—CMJ, and squat jump—SJ), speed (5-, 10-, and 35 m sprints), and agility (*t*-test) were assessed. Among body composition parameters, BF% presented the highest number of significant relationships with speed and agility, with the strength of correlations ranging from small (5 m sprint, r = 0.25) to large (35 m sprint, r = 0.52). The strongest correlation coefficient emerged between FFM and the 35 m sprint (r = −0.65). Significant correlations were found between vertical jump performance and the 35 m sprint (CMJ: r = −0.68; SJ: r = −0.69), followed by the *t*-test (CMJ: r = −0.35; SJ: r = −0.47). The hierarchical multiple regression model could explain 22% to 67% of the variance observed in agility scores and speed. BF% remained the most statistically significant negative predictor of all regression models. The CMJ remained a statistically significant positive predictor of the 35 m sprint (β = −0.581, *p* ≤ 0.01) after controlling for age and body composition. Integrating programs targeting lower-body power might be important to enhance speed and agility performance in youth soccer. On the other hand, future research based on multidisciplinary approaches to investigate the effects of nutritional strategies in reducing or preventing gains in BF% is still needed, which remained a significant predictor of sprint and agility performance in the final models.

## 1. Introduction

High-intensity actions, such as sprints, jumps, and changes of direction, characterize soccer [1]. At the elite level, previous research has reported short accelerations and linear sprints as frequent actions preceding goals and other decisive game situations [2]. The literature has described speed and agility capacities as distinguishing abilities for top-level soccer players [3], and there has been an emerging concern in the identification of conditioning programs designed to improve those capacities [4,5].

Consistently, lower-body power has been described as significant in supporting high-intensity action performance, including sprints and direction changes [6,7,8]. The literature has reported that higher strength levels are associated with success in lower-limb ballistic movements that accelerate body mass as quickly as possible, such as sprinting and change-in-direction actions [9]. Thus, lower-body power has emerged as an important physical attribute, frequently assessed through vertical jump tasks. Previous research among youth soccer players aged 16.2 ± 0.6 years found that eight weeks of additional lower-body power training promoted significant improvements in vertical jumps, speed, and agility tests [4]. In another study conducted with sports athletes aged between 12 and 15 years, four different lower-body power programs were reported as successful in improving vertical jumps and changes of direction performances [10]. Overall, the positive relationships between lower-body power, speed, and agility are well documented.

Meanwhile, age and body composition have also been significantly correlated with speed and agility performances. In youth soccer, age showed a significant and negative relationship with sprints and *t*-test times, indicating better performances among older players [11]. The same conclusion was reported in a systematic review, with the authors concluding that physical performance increases significantly with age [12]. On the other hand, there are reports of the detrimental effects of body fat percentage (BF%) on speed and agility capacities [11,13]. Additionally, research is available concerning the positive influence of fat-free mass (FFM) on physical performance [14,15].

Although previous research has targeted the influence of lower-body power on speed and agility tasks in youth soccer [4,16,17], the effects of age and body composition, particularly BF% and FFM, as possible confounder variables, have not been considered. This might lead to less accurate assessments, especially in youth populations. In the literature, some previous studies have targeted the relationships between body composition and lower-body power regarding speed and agility performance in youth soccer [6,16]. However, there is still a need for a detailed analysis of the influence of lower-body power tasks as a predictor of sprint and change in direction times after controlling for the effects of body composition parameters. Only one previous study was found in this matter [11]. However, BF% was the only body composition variable included in the analysis. Addressing these relationships is important in training young soccer players because it provides necessary insights into the factors influencing overall performance. This could allow coaches to develop more effective, personalized training programs to enhance players’ speed and agility. The main purpose of this study was to analyze, in detail, the variance in speed and agility performance explained by lower-body power tasks after controlling for age and body composition.

## 2. Materials and Methods

### 2.1. Study Design

This study is based on a cross-sectional assessment performed at the beginning of the sports season. The laboratory testing procedures were conducted in a physical fitness laboratory, while the field evaluations were implemented on a soccer training field. Testing sessions occurred during the morning period over five consecutive days. The procedures were monitored by trained staff and followed the Declaration of Helsinki. This study was approved by the Faculty of Human Kinetics Ethics Committee, CEIFMH Nº34/2021. The participation was voluntary, and informed consent was obtained from the youngsters’ legal guardians.

### 2.2. Participants

This study included 96 male soccer players, aged 16.1 ± 1.6 years, who played for the same club. Players’ training frequency ranged between four and five sessions per week, plus one match during the weekend at a regional level. Among the participants, 24 identified the left leg as preferred to kick the ball, while 72 reported the right leg as favored.

For this study, the optimal sample was calculated using G*Power 3.1 [18]. A priori hierarchical multiple regression analysis (seven predictors) determines a sample size of 80 participants to detect an interaction effect of 0.20 at the 0.05 significance level.

### 2.3. Body Composition

Height was measured to the nearest 0.01 cm using a stadiometer (SECA 213, Hamburg, Germany), while body composition was evaluated using hand-to-foot bioelectrical impedance analysis (InBody 770, Cerritos, CA, USA). The measurements were performed early in the morning. Participants stood on the platform, positioning their feet on specified spots and arms at approximately a 45° angle from their trunk. Throughout the measurements, participants wore only their underwear. Body mass, body fat percentage (BF%), and fat-free mass (FFM) were used for analysis.

### 2.4. Lower-Body Power

Lower-body power was assessed using the squat jump (SJ) and the countermovement jump (CMJ) [19]. Each protocol included four data collection trials separated by a 60 s rest period. The Optojump Next (Microgate, Bolzano, Italy) system was used for the measurement. Participants were incentivized to jump for maximum height in both tests, following three experimental trials to ensure proper execution. Participants returned to the starting position after each jump execution. In each protocol, the maximum height attained was retained for analysis. Afterward, a prediction equation, including jump height and body mass, were used to calculate the estimated peak power: Peak Power (W) = (60.7) × (jump height) + 45.3 × (body mass) − 2055 [20].

In the SJ protocol, participants began squatting with knees flexed to approximately 90º, holding for a three-count before jumping. The trial was repeated if the hip movement in the horizontal plane was detected.

The CMJ evaluation commenced with participants standing with their feet positioned hip-width to shoulder-width apart. Then, participants descended into a countermovement posture to a self-selected depth before executing a maximal-effort vertical jump. Throughout the movement, participants kept their hands placed on the hips to eliminate arm swing influence. Any deviation, such as hand movement or excessive knee flexion during the countermovement, led to trial repetition.

### 2.5. Speed

Speed was assessed through linear sprints performed at 5, 10, and 35 m, starting from a stationary position. Sprint time was recorded using the Witty-Gate photocells (Microgate, Bolzano, Italy) system. Two testing trials were performed, separated by a 2 min rest period, and the best score was used for analysis.

### 2.6. Agility

Agility was assessed using the *t*-test, which is a four-directional agility and body control test that examines the ability to change direction while maintaining balance rapidly and without losing speed [21]. Participants initiated by sprinting 9.14 m forward from a stationary position, then shuffled 4.75 m to the left side. Subsequently, participants shuffled to the right side 9.14 m and immediately shuffled 4.75 m back. Finally, participants ran backward until they passed the starting point. Test time was recorded using the Witty-Gate photocells (Microgate, Bolzano, Italy) system. Two testing trials were performed, separated by a 2 min rest period, and the best score was used for analysis.

### 2.7. Statistics

Descriptive statistics are resumed as means ± standard deviation. The Kolmogorov–Smirnov test was used to check the data’s normality. The relationships between body composition, lower-body power, speed, and agility were explored using the Pearson product–moment correlation coefficient. The strength of correlation was interpreted as follows: 0.1 < r < 0.3 (small), 0.3 < r < 0.5 (medium), and 0.5 < r < 1.0 (large) [22]. Then, hierarchical multiple regression analyses were conducted to investigate the amount of variance in speed and agility performance explained by lower-body power (entered in step 3) after controlling for age (entered in step 1) and body composition (entered in step 2). All analyses were performed using the IBM SPSS Statistics software 28.0 (SPSS Inc., Chicago, IL, USA). The significance level was set at 0.05.

## 3. Results

Table 1 resumes descriptive statistics for age, body composition, lower-body power, speed, and agility performances.

Table 2 and Table 3 summarize the correlation coefficients between age, body composition, lower-body power, speed, and agility variables. BF% presented the highest number of significant relationships with speed and agility among the body composition parameters. The strength of the correlations ranged between small (5 m sprint, r = 0.25) and large (35 m sprint, r = 0.52). The strongest correlation coefficient was observed between FFM and the 35 m sprint (r = −0.65, large). The analysis of lower-body power variables indicated significant negative relationships with speed and agility tests. The strongest correlation coefficients were found between vertical jump performances and the 35 m sprint (CMJ: r = −0.68, large; SJ: r = −0.69, large), followed by the *t*-test (CMJ: r = −0.35, medium; SJ: r = −0.47, medium).

Hierarchical multiple regression analyses were used to assess the ability of lower-body power (using the CMJ) to predict speed and agility performances after controlling for the influence of age and body composition (using stature, BF%, and FFM) (Table 4). The models as a whole were able to explain 29% (5 m sprint), 33% (10 m sprint), 67% (35 m sprint), and 23% (*t*-test) of the total variance observed in performance.

Age and body composition parameters alone were able to explain 28% (5 m sprint), 29% (10 m sprint), 61% (35 m sprint), and 22% (*t*-test) of the total variance observed in performance. BF% consistently remained a significant negative predictor in the final models, with higher values of BF% being associated with more time spent in speed and agility tests. In contrast, increased FFM was related to better performance scores in sprints and *t*-tests.

After controlling for age and body composition, the CMJ power explained an additional 3% and 6% of the variance in the 10 m and 35 m sprints, respectively. In the final models, age emerged as a statistically significant predictor of sprint performances, while CMJ power (β = −0.581, *p* ≤ 0.01) only remained a statistically significant predictor of the 35 m sprint.

## 4. Discussion

This study examined the relationships between lower-body power, age, body composition, speed, and agility performance among young male soccer players. The results indicated statistically significant relationships between age, BF%, FFM, lower-body power, and speed and agility tests. After controlling for age and body composition, CMJ power remained a significant predictor of the 35 m sprint performance. The models (age, body composition, and lower-body strength) could explain between 23% and 67% of the variance observed in speed and agility scores.

In the current study, BF% presented significant and positive relationships with speed and agility tasks, indicating that higher BF% is associated with longer sprints and changes in direction times. In contrast, the results reinforce the positive influence of FFM on speed and agility performances, with significant and negative correlations being observed. Our results support the hypothesis that excess body fat increases overall mass, potentially slowing quick movements. On the other hand, FFM provides the force, production, and power needed for fast movements and efficient direction changes, explaining the positive influence of FFM on muscular performance. In previous research on the developmental stages of soccer training, similar conclusions were reported regarding the negative effect of BF% on sprints and *t*-test performances [11,13]. Indeed, these results would be expected since the detrimental effect of BF% on sports performance has been apparent in tasks requiring body projection and rapid movement [15]. On the other hand, FFM has been strongly related to actions involving rapid skeletal muscle activation [23]. Thus, decreasing BF% and increasing FFM, including nutritional strategies and specific conditioning programs, should be considered throughout the players’ long-term development by coaches and their staff.

Meanwhile, significant negative correlations were also found between lower-body power, speed, and agility. Both the CMJ and the SJ presented the strongest relationships with the 35 m sprint and the *t*-test, suggesting that higher lower-body power levels are related to faster sprint and change in direction performance. Our results support the idea that power reflects the lower body’s ability to generate force rapidly, which is crucial for accelerating quickly and changing direction efficiently during sprints and agility tasks. Soccer players with greater lower-body power are believed to produce more force in shorter durations, enabling faster sprints and more effective directional changes [4]. A previous systematic review with meta-analysis concluded that increases in overall lower-body strength transfer positively to sprint performance [24]. In several sports contexts, higher lower-body power has been consistently associated with enhanced change in direction performance [7,8,11]. In a practical view, this information is critical because it underscores the significance of lower-body power in improving speed and agility performance among soccer players. In soccer, where split-second decisions and rapid movements are crucial, the capacity to accelerate rapidly and change direction efficiently greatly influences game outcomes.

When examining the models, the CMJ power appeared as a negative predictor of all tested variables after controlling for age and body composition parameters. Indeed, previous research has recommended high levels of lower-body power to enhance sprint capacity in soccer [16,25]. However, in the final models, the CMJ power only remained a significant predictor of the 35 m sprint performance, explaining an additional 6% of the observed variance. The results suggest the influence of lower-body power not only to accelerate (short sprints) but also to attain maximal speed. Indeed, the literature has reported maximal sprints as an important discriminator between elite players and their lower-division counterparts, including in youth soccer age categories [26]. Although there is evidence of significant correlations between vertical jump, speed, and agility performances in several sports contexts and populations [8,16,27], most of the published research has not considered the effects of age or body composition, which appear to be crucial variables to be contemplated, particularly during the players’ developmental stages.

The regression analyses of the present study showed that age and body composition variables could explain approximately 28%, 29%, and 61% of sprinting scores (5 m, 10 m, and 35 m sprint, respectively) and 22% of the *t*-test performance. Indeed, the literature has reported a significant influence of body composition parameters, particularly BF% and FFM, in tasks that require body displacement, such as jumping and running [15]. In research conducted on youth soccer players aged 7 to 19 years, the authors described that, in addition to age, BF% and FFM could be used as predictors of vertical jumps (CMJ and SJ) [28]. In another study, including 275 soccer players from different age groups, the authors found a large correlation between the CMJ height and FFM (r = 0.68, *p* < 0.01). Also, they reported a trend of increased CMJ performance during adolescence [14]. Adolescence includes the individual’s development toward maturity, which is strongly related to increased body size and physical performance [15]. For instance, in the previously mentioned study among youth soccer players [14], the authors explained the large gains in CMJ performance through increased FFM during adolescence. Besides, regarding sprint ability, differences between selected and non-selected players have also been described in youth soccer, with selected players significantly outperforming their peers [29].

According to the results, age remained a unique and significant predictor of short-distance sprints (5 and 10 m), probably reflecting its association with biological maturation. Although maturation is extremely individual in timing and tempo, the literature has described the age ranges between 10 and 22 years in boys as appropriate limits for normal variation in the onset and termination of adolescence [15]. Thus, the link between maturation and age must not be ignored when analyzing physical performance, particularly by the coaching staff involved in sports developmental stages.

This study presents some limitations that should be mentioned. First and foremost, players’ maturity status was not assessed. Although age might be associated with biological maturation, evaluating the maturity status would allow a deeper analysis regarding the relationships between body composition and physical performance. For instance, in a sample of 61 young male soccer players, sprint performance was related to age differences and persisted when body mass and FFM were statistically controlled. However, after the adjustment for age at peak height velocity, all between-group differences regarding speed disappeared [30]. Moreover, in team sports, there is evidence that suggests a selection bias towards more mature and larger individuals due to their increased physical capacities compared to their peers [31]. Thus, future studies on this topic, including adolescent participants, should include the maturity status assessment. Second, the data used is cross-sectional, and all players belonged to the same club; therefore, no inferences about causal relationships can be drawn, nor can the present findings be generalized to other populations. Including longitudinal data in future studies would allow a more detailed examination of the investigated variables.

Even so, the strengths of the current study should be highlighted, particularly by providing important insights into the role of body composition and lower-body power on speed and agility capacities among youth soccer players. FFM appears to be essential in decreasing sprint and changing direction times, while high levels of BF% should be avoided. However, future research designed to examine the effects of multidisciplinary approaches, including nutritional strategies, on reducing or preventing gains in BF% in youth soccer is still needed. On the other hand, the positive influence of lower-body power, particularly for agility tasks, has been demonstrated. Thus, besides focusing on skill acquisition, which is crucial during players’ developmental stages [32], specific programs to enhance lower-body power should be included in soccer training programs.

## 5. Conclusions

The results of the present study indicated statistically significant relationships between age, BF%, FFM, lower-body power tasks, and speed and agility tests. The detrimental effect of BF% on sprint and change in direction time has been shown, while FFM emerged as a significant predictor of speed and agility capacities. Although lower-body power presented significant correlations with speed and agility, the CMJ and SJ only remained substantial *t*-test predictors after controlling for age and body composition parameters. The results of the hierarchical regression analyses demonstrated that the models (age, body composition, and lower-body power) could explain between 22% and 67% of the variance observed in agility and speed scores. The results of this study emphasize the need for future research based on multidisciplinary approaches to investigate the effects of nutritional strategies on reducing or preventing gains in BF%, which remained a significant predictor of sprint and agility performance in the final models. On the other hand, the positive influence of lower-body power, particularly for agility tasks, underlines the importance of including specific programs to enhance lower-body power in soccer training sessions.

## Figures and Tables

**Table 1:** Descriptive statistics for youth soccer players’ age, body composition, lower-body power, speed, and agility performance (n = 96).

Variables	Mean ± Standard Deviation
Chronological age (years)	16.1 ± 1.6
Stature (cm)	174.3 ± 7.9
Body mass (kg)	65.0 ± 9.4
Body fat (%)	11.9 ± 5.4
Fat-free mass (kg)	57.1 ± 8.3
Countermovement jump (W)	2821.9 ± 615.9
Squat jump (W)	2774.8 ± 609.7
5 m sprint (s)	1.01 ± 0.07
10 m sprint (s)	1.75 ± 0.09
35 m sprint (s)	4.97 ± 0.33
*t*-test (s)	9.33 ± 0.52

**Table 2:** Correlation coefficients between age, body composition, speed, and agility performance of youth soccer players.

Variable	1.	2.	3.	4.	5.	6.	7.	8.	9.
1. CA	-	0.51 **	0.57 **	−0.18	0.64 **	0.25 *	0.04	−0.61 **	−0.28 **
2. Stature		-	0.68 **	−0.32 **	0.84 **	−0.05	−0.20	−0.52 **	−0.20
3. Body mass			-	0.22 *	0.89 **	0.12	−0.08	−0.40 **	−0.16
4. BF%				-	−0.25 *	0.25 *	0.33 **	0.52 **	0.33 **
5. FFM					-	−0.01	−0.24 *	−0.65 **	−0.32 **
6. 5 m sprint						-	0.63 **	0.13	0.05
7. 10 m sprint							-	0.51 **	0.34 **
8. 35 m sprint								-	0.52 **
9. *t*-test									-

CA (chronological age), BF% (body fat percentage), FFM (fat-free mass). * *p* ≤ 0.05; ** *p* ≤ 0.01.

**Table 3:** Correlation coefficients between lower-body power, speed, and agility performance of youth soccer players.

Variable	1.	2.	3.	4.	5.	6.
1. CMJ	-	0.94 **	−0.11	−0.31 **	−0.68 **	−0.35 **
2. SJ		-	−0.08	−0.32 **	−0.69 **	−0.47 **
3. 5 m sprint			-	-	-	-
4. 10 m sprint				-	-	-
5. 35 m sprint					-	-
6. *t*-test						-

CMJ (countermovement jump), SJ (squat jump). ** *p* ≤ 0.01.

**Table 4:** Summary of hierarchical regression analyses with lower-body power predicting speed and agility after controlling for CA and body composition of youth soccer players.

Variables	5 m			10 m			35 m			*t*-Test		
	Model I	Model II	Model III	Model I	Model II	Model III	Model I	Model II	Model III	Model I	Model II	Model III
	β	β	β	β	β	β	β	β	β	β	β	β
CA	0.310 **	0.565 **	0.589 **	0.554	0.443 **	0.496 **	−0.591 **	−0.276 **	−0.201 *	−0.292 **	−0.131	−0.095
Stature		−0.088	−0.086		0.092	0.094		0.032	0.035		0.155	0.157
BF%		0.328 **	0.366 **		0.382 **	0.465 **		0.412 **	0.529 **		0.353 **	0.411 **
FFM		−0.211	−0.052		−0.525 **	−0.175		−0.370 *	0.122		−0.252	−0.009
CMJ power			−0.188			−0.413			−0.581 **			−0.287
R^2^	0.096	0.279	0.285	0.004	0.294	0.325	0.349	0.613	0.672	0.085	0.220	0.234
SEE	0.070	0.064	0.064	0.093	0.079	0.078	0.271	0.213	0.197	0.504	0.475	0.473
*F* for change in R^2^	8.527 **	7.435 **	6.054 **	0.307	8.033 **	7.305 **	42.975 **	30.431 **	31.144 **	7.456 **	5.427 **	4.655 **

Model I: CA; Model II: CA, stature, BF%, FFM; Model III: CA, stature, body mass, BF%, FFM, CMJ power; CA (chronological age), BF% (body fat percentage), FFM (fat-free mass), CMJ (countermovement jump), SEE (standard error of the estimate), *F* (explains the incremental value of adding new predictors to the model). * *p* ≤ 0.05; ** *p* ≤ 0.01.

## Data Availability

The data supporting this study’s findings are available from the corresponding author upon reasonable request. The data are not publicly available due to privacy and ethical restrictions.
